# Process intensification for the continuous production of an antimicrobial peptide in stably-transformed *Sf-9* insect cells

**DOI:** 10.1038/s41598-022-04931-7

**Published:** 2022-01-20

**Authors:** Lukas Käßer, Maximilian Rotter, Luca Coletta, Denise Salzig, Peter Czermak

**Affiliations:** 1 Institute of Bioprocess Engineering and Pharmaceutical Technology (IBPT), Technische Hochschule Mittelhessen (THM) University of Applied Sciences, Giessen, Germany; 2 Faculty of Biology and Chemistry, Justus-Liebig-University Giessen, Giessen, Germany

**Keywords:** Biological techniques, Biotechnology

## Abstract

The antibiotic resistance crisis has prompted research into alternative candidates such as antimicrobial peptides (AMPs). However, the demand for such molecules can only be met by continuous production processes, which achieve high product yields and offer compatibility with the Quality-by-Design initiative by implementing process analytical technologies such as turbidimetry and dielectric spectroscopy. We developed batch and perfusion processes at the 2-L scale for the production of BR033, a cecropin-like AMP from *Lucilia sericata*, in stably-transformed polyclonal *Sf-*9 cells. This is the first time that BR033 has been expressed as a recombinant peptide. Process analytical technology facilitated the online monitoring and control of cell growth, viability and concentration. The perfusion process increased productivity by ~ 180% compared to the batch process and achieved a viable cell concentration of 1.1 × 10^7^ cells/mL. Acoustic separation enabled the consistent retention of 98.5–100% of the cells, viability was > 90.5%. The recombinant AMP was recovered from the culture broth by immobilized metal affinity chromatography and gel filtration and was able to inhibit the growth of *Escherichia coli* K12. These results demonstrate a successful, integrated approach for the development and intensification of a process from cloning to activity testing for the production of new biopharmaceutical candidates.

## Introduction

Antibiotic resistance has been described as an imminent global crisis by the World Health Organization due to the overuse of antibiotics and the lack of new antimicrobial drugs in the development pipeline^1^. In addition to the discovery of new small-molecule compounds^2^, alternative drug substances such as antimicrobial peptides (AMPs) have been gaining attention^3^ because they have multiple mechanisms of action^3–6^. However, the demand for such molecules would require efficient and continuous production processes.

To demonstrate a strategy for process development and intensification, we focused on the drug candidate BR033^7^, a cecropin-like AMP from the green bottle fly *Lucilia sericata*^8,9^ with a minimal inhibitory concentration (MIC) of 0.8 µM against *Escherichia coli*. AMPs are often toxic toward microbial production hosts so we considered insect cells as a more suitable platform, specifically the *Sf-9* cell line derived from *Spodoptera frugiperda*. This approach was selected because *Sf-*9 cells are ideal for the production of insect AMPs, including the addition of authentic posttranslational modifications, and are considered safe due to their evolutionary background^10–12^. We decided to generate a stably-transformed cell line expressing BR033 rather than using the baculovirus expression vector system (BEVS), which is often chosen for laboratory-scale and industrial applications^13^. Stable cell lines were considered more suitable despite the additional development time required because the BEVS relies on cell lysis, which not only affects protein glycosylation but also complicates continuous process modes such as perfusion^14^.

The production of recombinant proteins for clinical testing must comply with good manufacturing practice (GMP) and the Quality-by-Design (QbD) initiative, which requires strict process control^15–17^. Standard parameters as such as temperature, stirrer speed, pH and dissolved oxygen are straightforward to monitor and regulate^18–21^. However, cell-related parameters such as growth, viability, concentration and metabolic rates are more difficult to monitor in real time^22^. Culture-specific process analytical technology (PAT) tools as such as turbidimetry, dielectric spectroscopy (DS) and fluorimetry can increase process understanding and enable the control of key process events such as time of induction/infection, time of harvest, feeding, and bleeding^23,24^. However, critical process parameters (CPPs) must be identified when aiming to intensify the process by switching to a continuous production mode. Given the high oxygen demand of *Sf-9* cells^25^, the dissolved oxygen (DO) concentration is one such CPP and a suitable oxygenation strategy is required. Cell concentration and viability are also CPPs, so gentle cell retention, viability monitoring and robust turbidity control are also essential for process intensification.

## Materials and methods

### Cloning, cell culture and seed train

BR033 was expressed as a 60-kDa fusion protein comprising the honeybee melittin secretion signal, a His_6_ tag, the fluorescent protein tdTomato, a thrombin cleavage site and the C-terminal AMP sequence, under the control of the constitutive *OpIE2* promoter. The N-terminal His_6_-tag was used for protein purification, the fluorescent protein enabled quantification by fluorimetry, and the thrombin cleavage site was used to separate the AMP from the functional tags for activity testing. The plasmid containing the fusion construct and the bleomycin resistance gene was introduced into *Sf-9* TriEx cells (Merck, Darmstadt, Germany) in 20 mL Sf-900 II medium at a cell concentration of 1 × 10^6^ cells/mL (Thermo Fisher Scientific, Waltham, MA, USA) in a 100-mL baffled shake flask. The cells were transfected by lipopolyfection using 3 µL TransIT-Insect Transfection Reagent (Mirus Bio, Madison, WI, USA) and 1.5 µg DNA per milliliter of medium. The transfection reagent and DNA were mixed and incubated for 30 min before transfection according to the manufacturer’s instructions. A polyclonal cell culture was generated by selection in medium containing 500 µg/mL Zeocin (Invivogen, Toulouse, France). The cells were cultivated in 20 mL Sf-90 II medium in 100-mL baffled shake flasks at 28 °C without CO_2_ in a Celltron shaker (Infors, Basel, Switzerland) at 80 rpm with a 25-mm throw. The culture was split twice weekly and seeded in fresh medium at a concentration of 0.5 × 10^6^ cells/mL as previously described^26^.

### Bioreactor setup and online analytics

**Figure 1:**
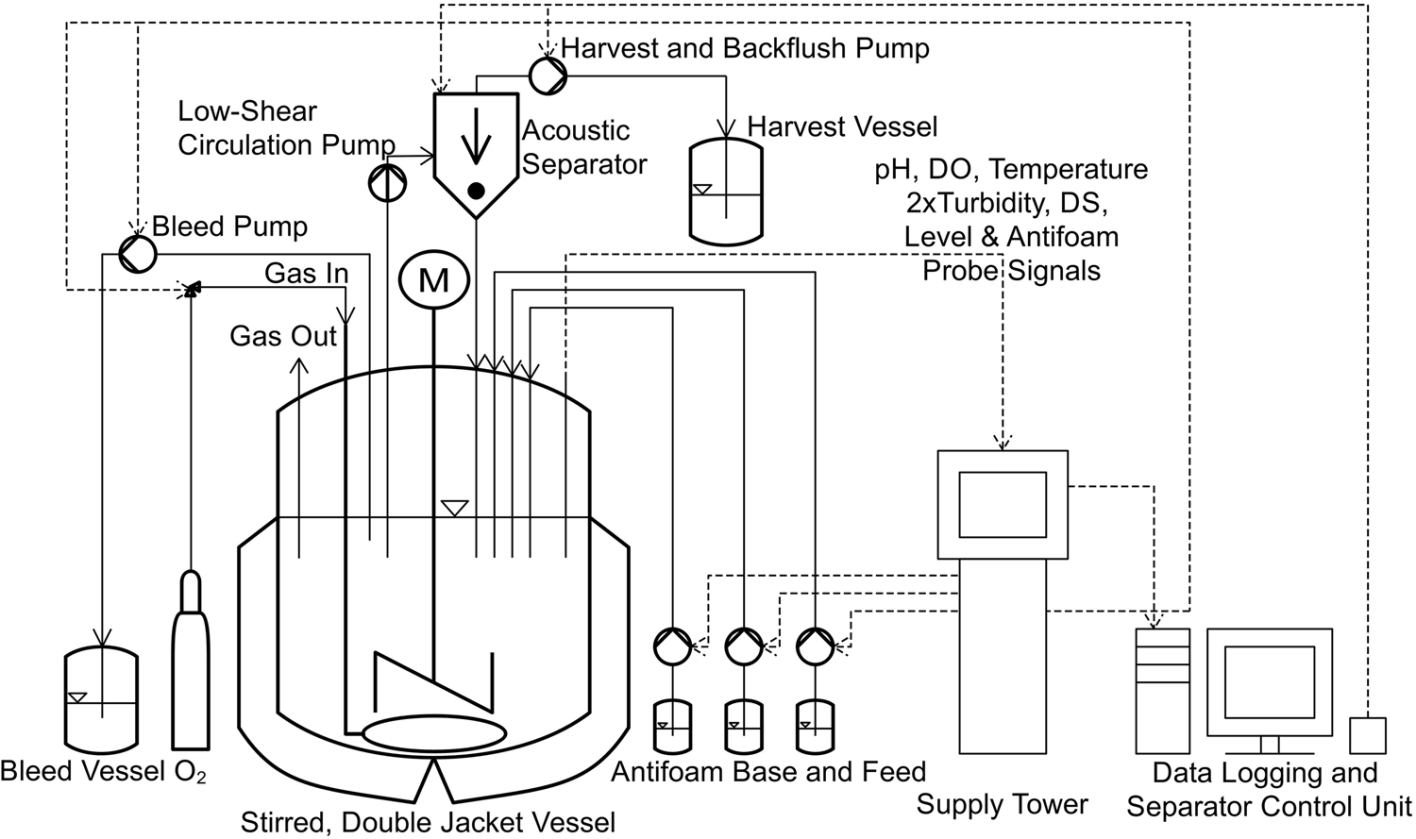
Flow diagram showing the process setup, with tubing represented by solid lines and data transmission represented by dashed lines.

We used a 5-L B-DCU bioreactor (Sartorius, Göttingen, Germany) with a working volume of 2 L. The setup, technical equipment and control loops are summarized in Fig. 1. The culture broth was stirred in a double jacket glass vessel at 100 rpm with a three-bladed pitched-blade impeller (78 mm diameter). Probes for pH and DO level (Hamilton, Bonaduz, Switzerland) as well as a PT100 temperature probe (Sartorius) were used for the control loops. The temperature in the double jacket glass vessel was maintained at 28 °C, a DO level above 40% air saturation was controlled by sparging with 100% O_2_ through a common ring sparger (0–0.5 vvm) and a pH of 6.3 was maintained by adding 1 M sodium hydroxide, with 1 M phosphoric acid added manually via a prefilled syringe if necessary.

The viable cell concentration was recorded with an Incyte DS probe (Hamilton, Bonaduz, Switzerland) and two different turbidity probes with 5-mm slits were used to monitor the total cell concentration. The ASD12-N (840–910 nm) turbidity probe (Optek, Essen Germany) was used for the bleed control loop whereas the EXcell 230 (880 nm) turbidity probe (Exner, Ettlingen, Germany) was used for comparison and to determine the residence time of cells in the separation unit due to its adjustable attenuation. The principles of DS and turbidimetry have been discussed^23,27^. For the perfusion setup, a PuraLev i30SU centrifugal pump (Levitronix, Zurich, Switzerland) was used to feed the external circulation loop to the acoustic separator unit (SonoSep, Vancouver, Canada).

A fixed flow rate of 24.75 mL/min was set for the circulation loop, whereas the cell mean retention time in the separation chamber was 1.51 ± 0.09 min (n = 3). The acoustic separator was set to 3 W and a duty cycle of 15 min with a backflush time of 5 s. A Masterflex L/S 300R peristaltic pump (Cole-Parmer, Vernon Hills, IL, USA) was used to harvest cell-free permeate and backflush the separation chamber. During backflushing, the harvest pump rotation was reversed and the flow was increased to 356 mL/min. After backflushing, the rotation direction was restored and harvesting resumed with the adjusted harvest rate. Because the backflush volume reduces the total harvest volume per unit time, the backflush volume was added, resulting in a harvest rate of 4.725 L/day and a net harvest rate of 2.1 L/day. Two level probes were used to regulate the feed and antifoam levels via separate pumps.

Regardless of the process mode, the setup was sterilized after assembly by autoclaving, and non-autoclavable parts were then assembled under aseptic conditions. Sf-900 II medium was filled into the vessel and heated to 28 °C overnight for sterility testing. The pH probe was calibrated before autoclaving and the DO, DS and turbidity probes were calibrated in fresh medium. The medium was inoculated with cells in the mid-exponential growth phase at a viability exceeding 90% and a starting concentration of 1 × 10^6^ cells/mL.

Perfusion cultivation was preceded by a batch phase, during which a perfusion rate of approximately one reactor volume per day (rvd) was established during exponential growth. The bleed control loop was set to the desired value (corresponding to the desired cell concentration), leading to enhanced growth with fresh medium and without bleeding until the setpoint was reached. The constitutive promoter in the expression vector ensured that the production of the fusion protein started immediately after inoculation.

### Offline analytics

During cultivation, cell concentration, viability, glucose and lactate concentrations, as well as fluorescence were analyzed after daily sampling. Cell concentration and viability were determined by trypan blue exclusion in a Neubauer counting chamber (Marienfeld, Königshofen, Germany), glucose and lactate were analyzed using the Biosen C (EKF Diagnostics, Barleben, Germany) according to the manufacturer’s instructions, and the fluorescence of 50-µL aliquots of supernatant was determined in a 96-well plate (Sarstedt, Nümbrecht, Germany) using a Cytation3 plate reader (BioTek, Winooski, VT, USA) with excitation and emission wavelengths of 554 and 581 nm, respectively, for the detection of tdTomato. The fusion protein concentration was calculated against a dilution series of tdTomato standards (OriGene Technologies, Rockville, MD, USA) in the cell culture medium, taking into account the different molecular masses.

### Downstream processing and activity testing

The culture broth or permeate was passed through a 0.22-µm bottle-top filter before immobilized metal affinity chromatography (IMAC) on an Äkta Start system fitted with a 1-mL HisTrap excel column (GE Healthcare, Chicago, IL, USA) using 750 mM imidazole buffer (20 mM Na_3_PO_4_, 0.5 M NaCl, 750 mM imidazole, pH 7.4). For thrombin cleavage, the purified protein was transferred into thrombin cleavage buffer (50 mM Tris, 0.1 M NaCl, pH 8) by gel filtration using a 50-mL Bio-Gel P-6 Gel Bio-Scale Mini cartridge (Bio-Rad Laboratories, Hercules, CA, USA) according to the manufacturer’s instructions. A thrombin-coupled Sepharose bead (TSB) suspension (BioVision, Milpitas, CA, USA) was then added at a ratio of 0.75 µL per µg protein and incubated at room temperature for 20 h for restriction. The suspension was then centrifuged for 1 min at 16,000×*g* and the supernatant containing the cleaved recombinant AMP was used for activity testing.

Activity testing was carried out in 96-well plates, with each well containing 40 µL of 2.5 × lysogeny broth and 10 µL of *E. coli* K12 cells (final OD_600nm_ = 0.01). 50 µL of phosphate buffered saline (PBS) were added for the negative control, 50 µL of bleocin (Merck) with a final concentration of 0.01 mg/L in PBS for the positive control, 50 µL of the cleaved and uncleaved recombinant fusion protein in thrombin cleavage buffer for testing. To rule out an influence of the buffer used, 50 µL of pure thrombin cleavage buffer were evaluated as a mock control. The inhibition of *E. coli* K12 cells was determined by measuring the OD600 every 10 min for 18 h.

### Consent for publication

All authors give their consent for the publication.

## Theoretical aspects of separation efficiency

Acoustic separation is based on the difference in density between cells and the culture medium^28,29^. In the acoustic resonance field, the primary and secondary radiation forces lead to loose aggregation at the pressure nodes, while the so-called Bernoulli force leads to string-like cell columns^30^. The efficiency of acoustic separation *E*_*Sep*_ is often defined (Eq. (1)) as the concentration of viable cells in the permeate scaled to the concentration of viable cells entering the separation chamber, equaling the concentration of viable cells in the stirred vessel assuming there is thorough mixing^31^.

1 $${E}_{Sep}=\left(1-\frac{{c}_{viable,permeate}}{{c}_{viable,reactor}}\right)\times 100 \%.$$

The justifiable assumption that the cell culture broth is an incompressible fluid leads to Eq. (2). Given a fixed loop flow rate, viable cell concentration in the reactor/loop, and viable cell concentration in the permeate flow, a lower permeate flow rate increases separation efficiency because fewer viable cells are flushed out the system via the permeate flow. In our system, the flow rates for harvest and recirculation were fixed and the adjusted separation efficiency *E*_Sep_^*^ was calculated using Eq. (3), considering the volumetric flow rates.

2 $${\dot{V}}_{recirculation}= {\dot{V}}_{loop}- {\dot{V}}_{permeate},$$

3 $${{E}_{Sep}}^{*}=\left(1-\frac{{c}_{viable,permeate}\times {\dot{V}}_{permeate}}{{c}_{viable,reactor} \times {\dot{V}}_{loop}}\right)\times 100 \%.$$

## Results and discussion

### Experimental overview

To ensure a sufficient oxygen supply, multiple batch cultivations were carried out with a microsparger or macrosparger to determine how oxygenation influences the cells and the oxygen control strategy. The microsparger interfered with the turbidity signal and led to solid foam buildup, therefore reducing cell viability. The macrosparger was selected for further experiments, and the oxygenation controller design and sparging rate were set to a maximum of 0.5 vvm. The batch process time was 96–142 h with fusion protein concentrations of approximately 1.5–2.5 mg/L. To intensify the process by transitioning from batch to continuous perfusion, we required robust turbidity control and careful monitoring of cell viability. We selected acoustic separation, which was used in an earlier perfusion process based on stable *Sf-*9 cells, allowing the production of secreted alkaline phosphatase for more than 1000 h with cell viability remaining above 75%^32^. It was also used to produce CBM-factor X with fixed-interval bleeding^33^. We established a standing acoustic wave in a low-shear external circulation loop for gentle cell retention combined with DS to monitor cell viability. To maintain a constant cell concentration during the continuous process, we established a turbidimetry control loop with automated bleeding.

### Turbidity control loop stability and PAT sensor data correlation

The stability of the turbidity control loop at different cell concentrations was tested in an initial perfusion stability run (PSR). The bleed controller was set to a direct proportional response with no dead band, switching on a peristaltic pump with a fixed rotation speed (flow = 17.1 mL/min) and pumping cell-containing culture broth into the bleed vessel. Level control was achieved using a simple electrical circuit. When contact with the liquid surface was broken, a direct proportional response was set. In this case, a second peristaltic pump with a fixed rotation speed (flow = 17.1 mL/min) was switched on, pumping fresh medium into the reactor.

**Figure 2:**
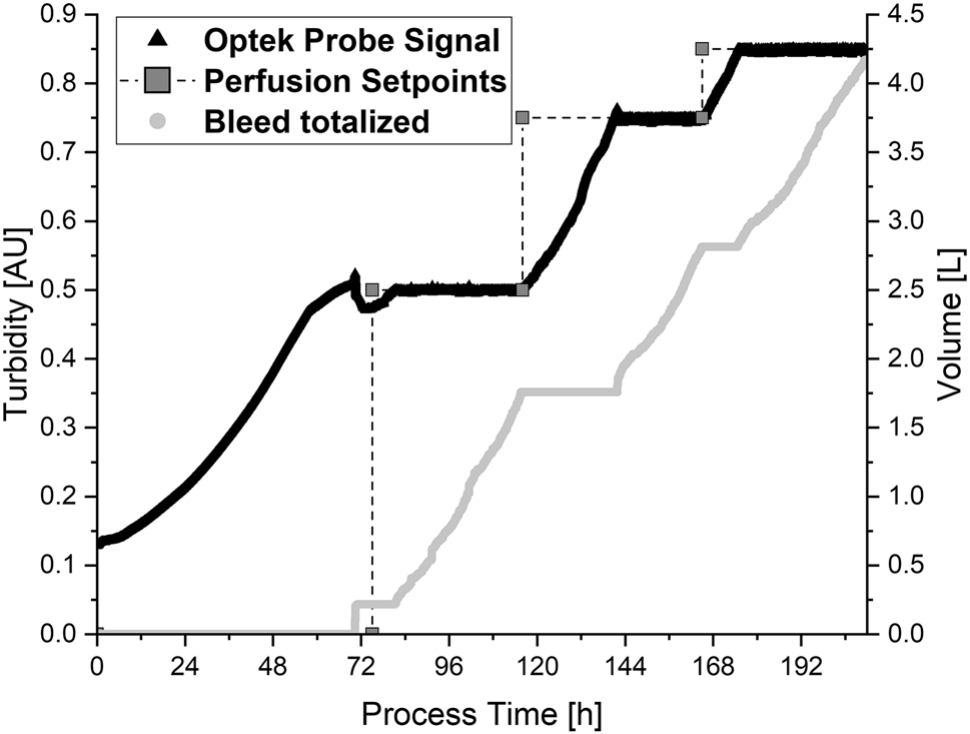
The turbidity signal in arbitrary units (AU, black triangles) during the control loop test run and the target setpoints in steps (dark gray squares). The corresponding bleed volume (light gray circles) was used to calculate growth rates during the cultivation phases with fixed cell concentrations.

The reactor was inoculated with 1 × 10^6^ cells/mL (129.6 mAU) and the control loop was activated with a setpoint of 500 mAU when the target turbidity of 500 mAU was reached. After a brief dip, the turbidity was controlled within a range of 494.8 ± 8.2 mAU over a period of 41 h (Fig. 2). The bleed rate and therefore also the growth rate was 0.0219 1/h. At a process time of 116 h, the setpoint was increased to 750 mAU, and this was reached after a process time of 142 h (∆_t_ = 26 h). The turbidity was controlled within a range of 747.2 ± 1.8 mAU for 23 h with a bleed and growth rate of 0.0196 1/h. As expected, the bleed and growth rate at this setpoint was close to the rate at the first setpoint.

The setpoint was increased again to 850 mAU at a process time of 165 h, and this was reached after a process time of 174 h (∆_t_ = 9 h). The turbidity was controlled at a value of 847.2 ± 1.7 mAU over 39 h and the bleed and growth rate was 0.0199 1/h. All calculated bleed/growth rate values fell within the range of 0.0205 ± 0.0013 1/h, which is lower than the equivalent values during batch cultivation. The PAT-based bleeding strategy therefore enabled the strict control of turbidity at all three setpoints.

**Figure 3:**
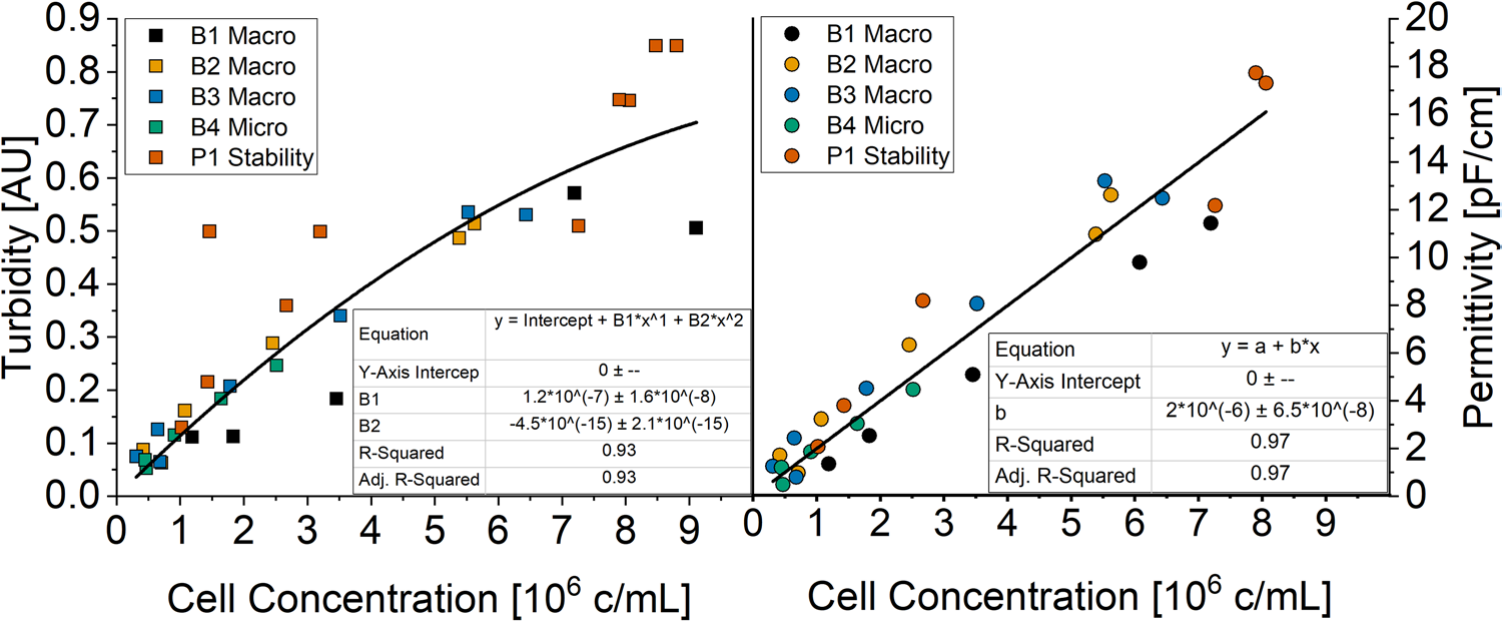
Cultivation data used to calculate the correlation between viable cell concentration and turbidity or permittivity. B1–3 = batch cultivations with macrosparger, B4 = batch cultivation with microsparger, P1 = perfusion stability run.

The batch data and PSR data were used to calculate correlations between the turbidity and DS signals and cell concentration, as shown before with *Drosophila melanogaster* Schneider 2 (S2) cells^23^. The model describing the relationship between turbidity and viable cell concentration (Fig. 3) allowed the cell concentration to be stabilized, whereas the correlation with the DS signal provided information about cell viability and can be used to calculate the growth rate during the growth phase. A negative deviation of the DS signal from the turbidity signal indicates a drop in viability. The growth rate calculated from the DS signal is more accurate the when derived from the turbidity signal because it is less susceptible to debris, bubbles and dead cells^18,23^. A second-order polynomial model was used for the correlation of the turbidity signal, whereas a linear model was suitable for the correlation of the DS signal as previously described^34^.

### Process intensification by switching to perfusion mode

**Figure 4:**
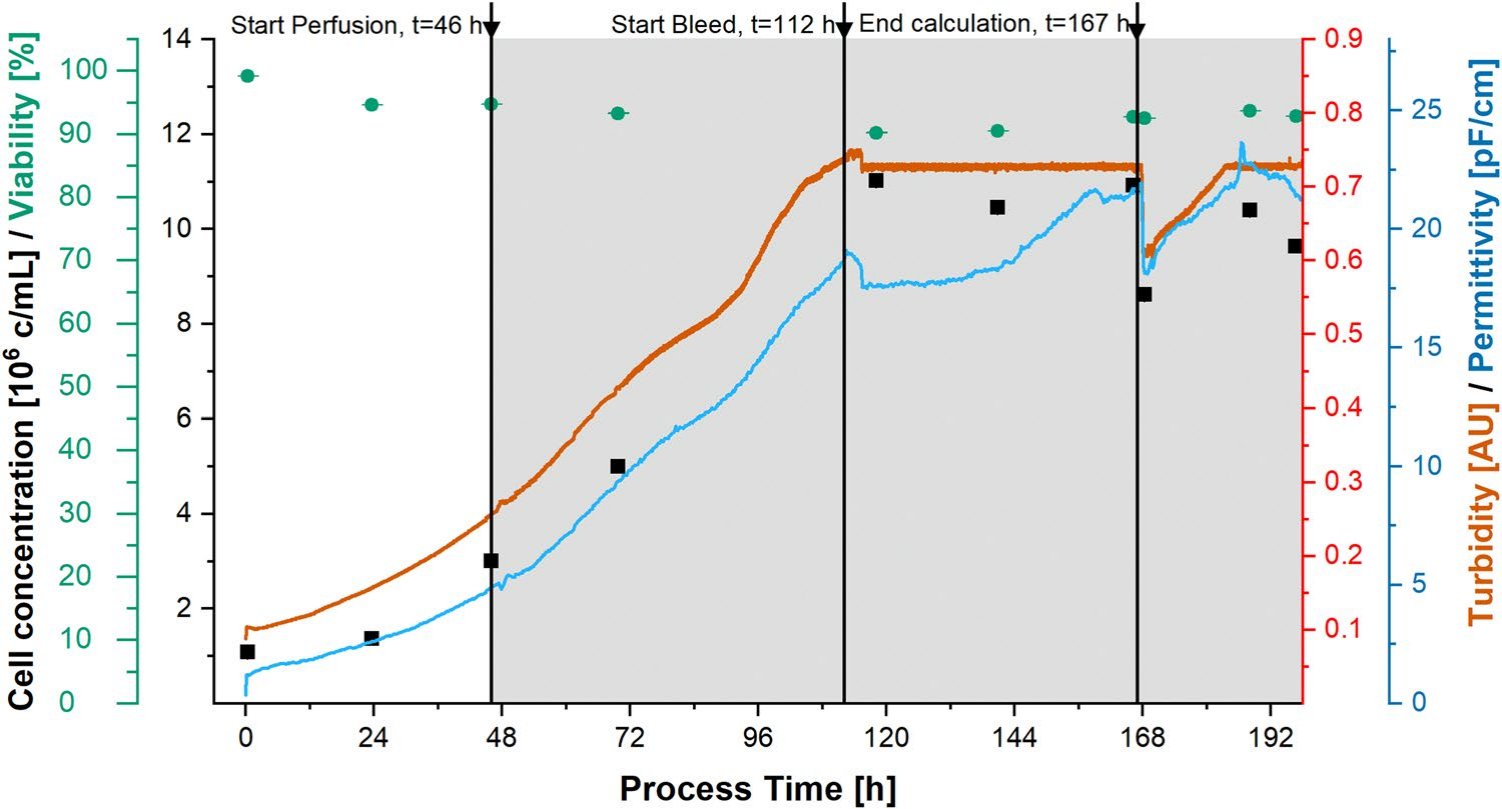
Perfusion process data from offline (left axes) and online (right axes) measurements. Acoustic separation and harvesting were started at t = 46 h. The turbidity setpoint was reached at t = 112 h, all process parameters were calculated from this point for the subsequent 55-h period.

For perfusion-mode cultivation, the reactor was inoculated at a concentration of 1 × 10^6^ cells/mL, and the bleed controller, harvest pump and separator were activated after 46 h. The target cell concentration was 1 × 10^7^ cells/mL and a corresponding AU setpoint of 730 mAU was calculated using the model derived from the batch cultivation and PSR. The AU setpoint was reached at a process time of 112 h, and automated bleeding then started at a rate of 49.6 mL/h (Fig. 4). Bleeding continued until a process time of 167 h, when a disturbance was simulated by manual bleeding to test controller robustness. The control worked: the 730 mAU setpoint was reached once again 188 h after inoculation and was maintained until the fresh medium was exhausted. When the turbidity was 730 mAU (calculated for 1 × 10^7^ cells/mL) offline analysis during perfusion showed a concentration of 1.1 × 10^7^ ± 3.0 × 10^5 ^cells/mL with a viability of 91.1% ± 1.4% (both n = 3 over 55 h), which is close to the target concentration and confirms the accuracy of the model. During the 198-h cultivation, the cell viability remained at least 90.5%. The DO control loop worked well because the DO concentration did not drop below the critical level and we did not detect lactate accumulation, which is caused by oxygen limitation^35^. The glucose concentration was 6.6–7.6 g/L, confirming the absence of glucose limitation. Table 1 summarizes the process parameters for the batch and perfusion processes.

**Table 1:** Mean growth rate µ, production rate rp, cell-specific production rate qp, space–time yield STY and maximum product concentration Cpmax for the batch processes (n = 3) and the 55-h steady-state phase of the perfusion process with a macrosparger. Literature data for stable Sf-9 cell lines converted to the units used herein are shown in bold.

	This article		Literature		
	Batch (n = 3)	Perfusion	Batch	Perfusion	Sources
µ (1/h)	0.0271 ± 0.0005	0.0248	**0.013–0.037**	**0.015–0.025**	^35,38–41^
*r*_*p*_ (mg/h)	0.0327 ± 0.0144	0.0917	n.a	**0.375**	^33^
*q*_*p*_ [mg/(c_cell_ × h)]	2.49 × 10^–12^ ± 3.67 × 10^–13^	4.17 × 10^–12^	n.a	**8.3 × 10**^**–12**^	^33^
*STY* [mg/(L × h)]	0.0173 ± 0.0076	0.0459	n.a	**0.25**	^33^
*C*_*pmax*_ (mg/L)	1.9887 ± 0.5093	0.9077	n.a	**5.0**	^33^

**Figure 5:**
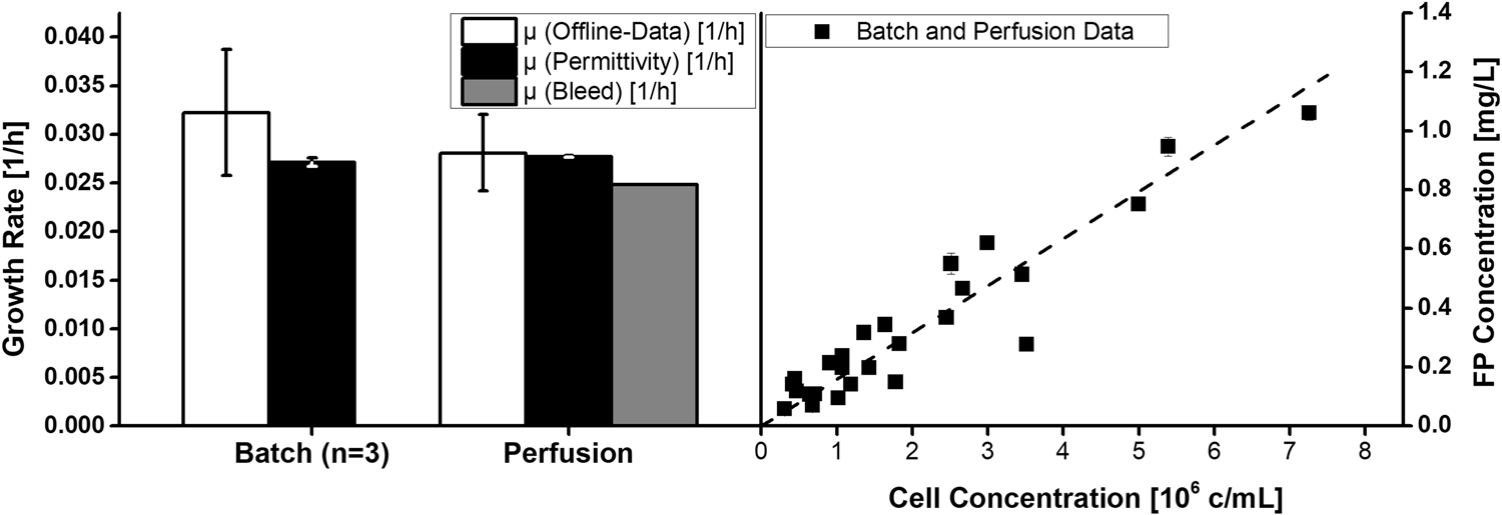
Differences in the growth rate µ based on the calculation input data (left) and calculated fusion protein concentration for the exponential phase of all cultivations over the corresponding offline cell concentration (right).

Growth in both process modes was in the upper range of values reported in the literature, indicating that our process control and process parameter settings were appropriate. Figure 5 (left side) shows a significantly higher error when the growth rate *µ* was calculated using offline data rather than DS data, but there were no large deviations in *µ* based on DS data from different runs. This consistency, in addition to the model derived from the batch and PSR runs, once again shows that DS is a very powerful PAT tool for the online monitoring of viable cell concentration. During perfusion at a fixed turbidity, µ was calculated from the bleed rate under the assumption of ideal separation efficiency, which is reasonable since the separation efficiency observed during this phase of the process was close to ideal (> 97%). Compared to the batch processes and exponential growth phase before reaching the turbidity setpoint, *µ* was slightly lower during this phase of the process (Table 1), which is consistent with the literature. We assume cell growth during perfusion is affected by the circulation and retention time in the separation device due to changes in temperature and oxygen supply. Even though retention times are generally short and the statement is consistent with our retention time of ~ 1.5 min^36^, an increase in temperature of 5–8 °C was observed due to the separator power input in a CHO perfusion process with a similar net harvest rate^31^. Furthermore, the growth of insect cell lines becomes slower without a sufficient supply of autocrine growth factors^37^. This may reflect the continuous removal of such factors by harvest and bleeding, plus the dilution of the remaining growth factors with fresh medium.

Process intensification from batch to perfusion led to a 180% increase in the production rate (*r*_*p*_), a ~ 70% increase in cell-specific productivity (*q*_*p*_), and a 170% increase in the space–time yield (*STY*). A similar process with acoustic separation for the production of a CBM-factor X fusion protein in stably-transformed *Sf-9* cells achieved a *q*_*p*_ value twice as high as ours, probably reflecting the use of a monoclonal high-producer cell line^33^. Another monoclonal high-producer *Sf-9* cell line achieved a *q*_*p*_ value tenfold higher than our polyclonal culture^42^. The steady-state cell concentration also has a major impact on *r*_*p*_ and *STY*, with higher cell concentrations achieving better performance because protein expression is directly associated with the viable cell concentration, as shown in the right-hand panel of Fig. 5^33^.

### Cell damage and separation efficiency

Separation efficiencies *E*_*Sep*_ and *E*_Sep_^*^ were calculated for the PSR and the main perfusion run. The influence of harvest flow, circulation flow and separator power for *Sf-*9 cells in an BioSep ADI 1015 ultrasonic filter have been investigated before, revealing higher separation efficiencies at high power settings (≥ 3 W) with low circulation and harvest flow rates^43^.

**Figure 6:**
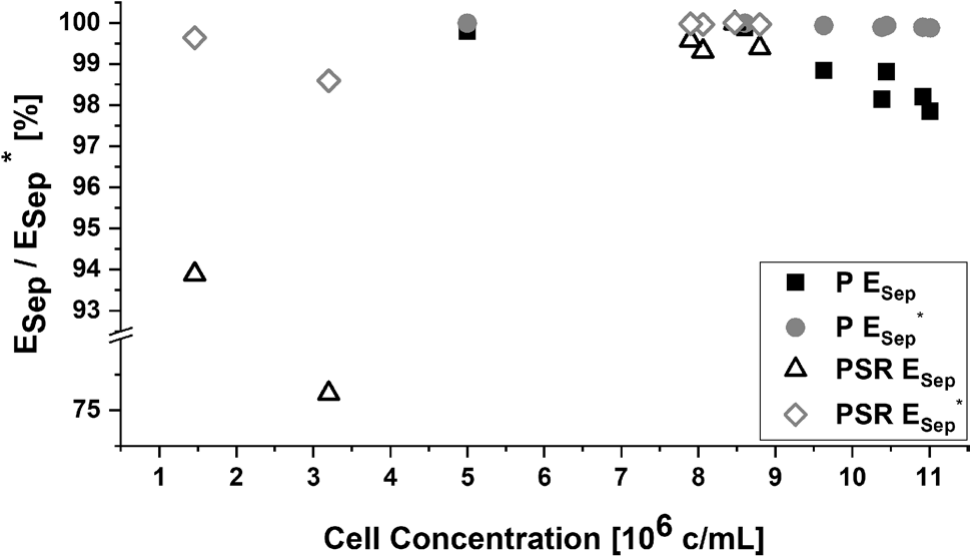
ESep and ESep* values calculated from the perfusion stability run (PSR) and perfusion run (P).

Cell viability in a previous study was shown to decrease rapidly in serum-free medium at recirculation flow rates of 10 and 16.7 rvd achieved using a peristaltic pump, whereas viability was unaffected at circulation rates of 17.5 rvd when cells were cultivated in medium containing 1% fetal bovine serum^33^. With a moderate power input (3 W) and a harvest rate of 4.725 L/day, we observed no cell damage in serum-free medium when the broth was circulated using a low-shear centrifugal pump at a flow rate of 17.8 rvd. A harvest rate of ~ 2.4 rvd was reported to achieve an *E*_*Sep*_ > 95% in a similar *Sf-*9-based perfusion process^43^, and we likewise observed *E*_*Sep*_ and *E*_Sep_^*^ values > 97% at the same harvest rate as long as the cell concentration was greater than 5 × 10^6 ^cells/mL (Fig. 6). Bearing in mind the definition of E_Sep_^*^, separation efficiency is affected not only by the circulation flow rate and cell concentration, but also by the ratio of harvest rate to circulation rate. A ratio < 0.5 tending to 0 pulls the loosely aggregated cells toward the recirculation outlet of the separation chamber, whereas a ratio > 0.5 tending to 1 facilitates the discharge of cells through the harvest outlet by reducing sedimentation speed or even dragging the trapped cells against the gravity field.

### Downstream processing, cleavage and activity testing

The culture supernatant was applied to an IMAC column, which increased the fusion protein concentration by 90-fold. The first three elution fractions contained the 60-kDa fusion protein with a recovery comparable to previous reports^44^. Size exclusion chromatography (SEC) resulted in a 1.6-fold dilution. The recovery of protein following the IMAC, SEC and cleavage steps, and the overall recovery from culture broth to post-cleavage is summarized in Table 2.

**Table 2:** Percentage recovery for individual purification steps and the complete purification process.

Purification step	IMAC	SEC	Thrombin cleavage	Total
Recovery	93%	87%	93%	75.2%

SDS-PAGE analysis of the IMAC fractions revealed that ~ 30% of the fluorescence could be traced to premature cleavage of the fusion protein in the supernatant and/or errors during protein synthesis which affect ~ 15% of the protein molecules^45^. We therefore included a correction factor of 0.7 for all concentration calculations based on fluorescence readings. To confirm the activity of recombinant BR033, the fusion protein was tested for its ability to inhibit *E. coli* K12. Thrombin cleavage buffer without the recombinant protein (mock) had no significant effect (data not shown) in comparison with the negative control. The positive control inhibited the growth of *E. coli* K12 completely, whereas the inhibitory activity of the uncleaved fusion protein was 3.4 ± 1.3%.

BR033 with an amidated C-terminus has a MIC of 0.8 µM^8^. The amidated C-terminus enhances the activity of BR033 by stabilizing the α-helix^46^. Additionally, thrombin cleavage leaves behind glycine and serine residues at the N-terminus, which can affect the overall charge and therefore the activity of the AMP^47^. After thrombin cleavage, we tested BR033 at concentrations of 0.8 and 1.35 µM, revealing inhibitory activities of 21.8 ± 1.1% and 28.7 ± 3.1%, respectively. This confirms that we were able to produce soluble and active recombinant BR033 with the perfusion process described in this work.

## Conclusion and outlook

To the best of our knowledge, this is the first time that a soluble AMP fusion protein has been produced continuously in stably-transformed *Sf-*9 cells and its activity has been confirmed following release from the fusion protein. In contrast to earlier perfusion processes with *Sf-9* cells, we established a PAT-based system for the automated control of turbidity and level at a constant harvest rate. Acoustic separation was efficient without significant physical interactions, eliminating the problem of fouling that is prevalent in membrane-based separation processes. Furthermore, the use of a low-shear pump for circulation ensured that the cells remained viable. Perfusion achieved a high growth rate at a relatively high cell concentration compared to previous reports. It may be possible to improve the yield further by increasing the viable cell concentration at the perfusion setpoint or by isolating a monoclonal high-producer cell line with a higher cell-specific productivity^42,48^. For example, a seven-fold increase in yield was achieved by selecting a monoclonal high-producer S2 cell line^49^. Combined with a higher cell concentration, it should be possible to increase yields by more than an order of magnitude. Adjustments to the harvest have been shown to improve the yield per liter^50^ and may also increase the yield per unit time because growth factors produced by *Sf-*9 cells, which promote cell growth and inhibit apoptosis^51^, remain in the culture broth for longer. A prolonged perfusion process also reduces the setup time compared to repeated batch processes.

Cells die when trapped in foam and do not contribute to protein production, which would reduce cell viability in long-term processes. Switching to a bubble-free oxygenation strategy could enhance process control by eliminating foam and also removing a source of interference with inline measurements. The viable cell concentration could also be controlled by integrating the DS signal for the bleed strategy. DS has been implemented in seed trains for multiple processes modes with PER.C6, CHO (C5.23SFM1) and SP2/0 cells^22,50,52,53^. In addition to control loops measuring viable cell concentration, or the determination of viability in combination with turbidimetry, DS could also be used to examine culture aging in long-term perfusion processes. To enhance AMP activity, the most promising approach would be to amidate the C-terminus of the recombinant AMP after purification, because amidated AMPs tend to have the lowest MICs^8^. The MICs of C-terminal amidated α-helical peptides are lower than those of C-terminal carboxylated α-helical peptides^54^. Taken together, our results show that we were able to produce soluble, active BR033 in considerable amounts. The PAT-based continuous process is suitable for the production of novel drug candidates and allows the production of active proteins with posttranslational modifications when lytic processes are unsuitable.

## Data Availability

The datasets generated during and/or analyzed during the current study are available from the corresponding author on reasonable request.

## Abbreviations

AMP: Antimicrobial peptide
*Sf-*9: *Spodoptera frugiperda* 9
BEVS: Baculovirus expression vector system
GMP: Good manufacturing practice
QbD: Quality by design
PAT: Process analytical technology
DS: Dielectric spectroscopy
rvd: Reactor volume per day
IMAC: Immobilized metal affinity chromatography
TSB: Thrombin Sepharose beads
OD: Optical density
PBS: Phosphate-buffered saline
PSR: Perfusion stability run
STY: Space–time yield
MIC: Minimal inhibitory concentration
DO: Dissolved oxygen
